# Sustained Reduction in Third-generation Cephalosporin Usage in Adult Inpatients Following Introduction of an Antimicrobial Stewardship Program in a Large, Urban Hospital in Malawi

**DOI:** 10.1093/cid/ciaa162

**Published:** 2020-02-15

**Authors:** Rebecca Lester, Kate Haigh, Alasdair Wood, Eleanor E MacPherson, Hendramoorthy Maheswaran, Patrick Bogue, Sofia Hanger, Akuzike Kalizang’oma, Vinothan Srirathan, David Kulapani, Jane Mallewa, Mulinda Nyirenda, Christopher P Jewell, Robert Heyderman, Melita Gordon, David G Lalloo, Rachel Tolhurst, Nicholas A Feasey

**Affiliations:** 1 Malawi Liverpool Wellcome Trust Clinical Research Programme, Blantyre, Malawi; 2 Liverpool School of Tropical Medicine, Liverpool, United Kingdom; 3 Health Protection Team, Public Health England, Bristol, United Kingdom; 4 Institute of Population Health Sciences, University of Liverpool, Liverpool, United Kingdom; 5 Conflict and Health Research Group, Kings College London, London, United Kingdom; 6 Imperial College Healthcare National Health Service Trust, London, United Kingdom; 7 Division of Infection and Immunity, University College London, London, United Kingdom; 8 Northumbria Healthcare National Health Service Trust, Newcastle, United Kingdom; 9 College of Medicine, University of Malawi, Blantyre, Malawi; 10 Department of Medicine, Queen Elizabeth Central Hospital, Blantyre, Malawi; 11 Adult Emergency and Trauma Centre, Queen Elizabeth Central Hospital, Blantyre, Malawi; 12 Centre for Health Informatics, Computing and Statistics, Lancaster University, Lancaster, United Kingdom; 13 Institute of Infection and Global Health, Liverpool, United Kingdom

**Keywords:** antimicrobial resistance, antimicrobial stewardship, Africa south of the Sahara

## Abstract

**Background:**

Third-generation cephalosporins (3GC) remain the first-choice empiric antibiotic for severe infection in many sub-Saharan African hospitals. In Malawi, the limited availability of alternatives means that strategies to prevent the spread of 3GC resistance are imperative; however, suitable approaches to antimicrobial stewardship (AMS) in low-income settings are not well studied.

**Methods:**

We introduced an AMS intervention to Queen Elizabeth Central Hospital in Blantyre. The intervention consisted of a prescribing application for smartphones and regular point-prevalence surveys with prescriber feedback. We evaluate the effects of the intervention on 3GC usage and on the cost of providing antibiotics. Using a thematic analysis of semi-structured interviews and participant observations, we additionally evaluate the acceptability of the stewardship program.

**Results:**

The proportion of antibiotic prescriptions for a 3GC reduced from 193/241 (80.1%) to 177/330 (53.6%; percentage decrease, 26.5%; 95% confidence interval, 18.7–34.1) with no change in the case-fatality rate. The cost analysis estimated an annual savings of US$15 000. Qualitative research revealed trust in the guideline and found that its accessibility through smartphones helpful to guide clinical decisions. Operational health-system barriers and hierarchal clinical relationships lead to continued reliance on 3GC.

**Conclusions:**

We report the successful introduction of an antimicrobial stewardship approach in Malawi. By focusing on pragmatic interventions and simple aims, we demonstrate the feasibility, acceptability, and cost savings of a stewardship program where resources are limited. In doing so, we provide a suitable starting point for expansions of AMS interventions in this and other low-income settings.

## Teaser

An antimicrobial stewardship program, consisting of a prescribing guideline and point-prevalence surveys with prescriber feedback, was introduced at Queen Elizabeth Central Hospital in Malawi. The program was effective in reducing third-generation cephalosporin usage and was potentially cost-saving for the hospital.

Infection with antimicrobial resistant (AMR) pathogens is associated with high morbidity and mortality for patients and a significant economic burden on health systems [1, 2]. In sub-Saharan Africa (sSA), the prevalence of AMR in key pathogens is amongst the highest in the world [3] and the mortality burden from drug-resistant infections is predicted to be huge [4]. Interventions that reduce excessive antimicrobial usage have been shown to reduce AMR [5], and the optimization of antimicrobial usage is a key World Health Organization target. However, antimicrobial stewardship (AMS) programs are typically tailored to high-income settings, frequently incorporating specialist infection liaison services, advanced microbiological diagnostics, and a restrictive antibiotic formulary [5, 6]. Few hospitals in sSA have AMS programs in place [7], and a better understanding of the ideal components of an effective and acceptable AMS intervention in a resource-limited setting is needed.

Ceftriaxone, a parenteral third-generation cephalosporin (3GC), has long been the antibiotic of choice in many sSA hospitals, with its once-daily dosing regimen and broad spectrum of activity making it a useful and convenient choice in settings where diagnostics and nursing capacities are limited [8, 9]. In sSA, the prevalence of 3GC resistance amongst key bloodstream pathogens is high [10], and widespread reliance on ceftriaxone has likely been a major driver of this class-level resistance [11–13].

In Malawi, sentinel surveillance from patients presenting to Queen Elizabeth Central Hospital (QECH) in Blantyre has demonstrated a rapid rise in 3GC resistance amongst bloodstream Enterobacterales, occurring contemporaneously with the widespread roll-out of ceftriaxone [14]. A limited availability of alternative antibiotics means that reducing class-level resistance to cephalosporins and preventing further AMR transmission is of critical importance. AMS is a key strategy for achieving this [15], yet little is known about either the antibiotic prescribing practices at QECH or what constitutes deliverable, acceptable, and sustainable interventions and targets for an AMS program in a resource-poor hospital.

The aim of this work was to establish and evaluate a formal AMS program on the adult medical wards at QECH. We conceived and introduced a locally appropriate antibiotic guideline and evaluated its impact on ceftriaxone usage and the cost of antibiotic provisions. Incorporating qualitative methodology, we additionally aimed to understand the barriers to and enablers of our stewardship approach.

## METHODS

### Setting

Malawi is a low-income country with a low health-care expenditure (~3% gross domestic product) [16]. QECH is a 1300-bed unit and is the only hospital providing free inpatient care in Blantyre. There are 200 adult medical beds, and ~70% of adult inpatients are living with human immunodeficiency virus (HIV) [17]. Quality-assured blood cultures and cerebrospinal fluid analyses are provided by the Malawi-Liverpool Wellcome Trust Clinical Research Programme, but there is no formal microbiology liaison service. Prior to this study, there were no formal AMS activities and no specific antibiotic guidelines.

### Study Design

#### Overview

The stewardship program had 3 phases: a preimplementation prescribing survey, designed to identify stewardship targets (January 2016); the implementation of an antibiotic guideline (June 2016); and 2 postimplementation prescribing surveys (January 2017 and January 2018). Point-prevalence surveys (PPS) with feedback to prescribers began at the start of the implementation phase and continued for 28 months. The study methods are described below, as well as in more detail in the Supplementary Material.

#### Antibiotic-prescribing Surveys

Inpatient records were reviewed to collect data on key stewardship quality indicators. The preimplementation survey was carried out by R. L., D. K., and A. K. Then, R. L. drafted the antibiotic guideline. Postimplementation surveys were carried out by a doctor who was not involved in the implementation phase (K. H.). We adapted standardized stewardship quality indicators to suit local practice [18], focusing on measurable targets, such as a 48-hour antibiotic review and the durations of antibiotic prescriptions (Supplementary Methods; Table 1).

#### Stewardship Intervention

A series of multidisciplinary team meetings were held to establish the AMS program, convening clinicians, nursing managers, hospital directorship, pharmacists, and microbiologists. All key decisions involved senior clinicians active at QECH. Local antimicrobial susceptibility trends, antibiotic availability, and national guidelines were reviewed and used to design a consensus-based antibiotic guideline that was reviewed by all senior clinicians in the departments. In Malawi, the burden of severe bacterial infection is high, and it is critical that AMS does not restrict access to potentially lifesaving therapy [19]. Our guideline therefore focused on the importance of recognizing and treating sepsis and on a 48-hour antibiotic step-down.

The guideline was distributed using booklets and posters and as a smartphone antibiotic application, Microguide (http://www.microguide.eu/). Microguide allows prescribers to access hospital-specific guidelines on their mobile devices or the hospital intranet. The QECH version can be viewed at https://viewer.microguide.global/QECH/ADULT4.

#### Point-prevalence Surveys

An average of 2 to 3 PPS were carried out per month. In each survey, 2 numerators were defined: the number of patients on ceftriaxone and the number of patients on at least 1 antibiotic. All adult medical inpatients whose notes were available at the time of the survey were included, and results from each PPS were presented to Department of Medicine meetings the following day. These meetings are compulsory for all cadres of clinicians and medical students working within the department. Results were presented verbally by a study doctor who was clinically active on the medical wards. Attendees were informed what proportion of their patients were on ceftriaxone and what proportion were on at least 1 antibiotic, and were given a reminder to review their prescriptions.

#### Qualitative Methods

Semi-structured interviews and observations with clinicians were carried out throughout the study (Supplementary Methods). Interviewees were purposively sampled based on their role in prescribing antibiotics, and observations were conducted on medical ward rounds and in morning meetings. Transcripts and field notes were imported into the Nvivo software package (QSR International Pty Ltd, Version 11, 2015) and a thematic analysis was conducted.

#### Cost Analysis

We explored the impact of the stewardship intervention on the direct health-provider costs of supplying antibiotics at QECH. First, we used data captured in the 3 prescribing surveys to estimate the total cost of all antibiotics given to participants in each survey. We divided this total cost by the total number of participants in each of the 3 surveys to estimate the average cost per participant. Secondly, we modelled how the stewardship intervention would impact the mean cost per patient and the total annual cost of providing antibiotics to those admitted to the 3 medical wards (male medical; female medical; tuberculosis [TB] ward). We also estimated the annual cost of implementing the intervention (Supplementary Table 2).

#### Statistical Analysis

Stewardship indicators from antibiotic surveys were described as proportions, and comparisons between pre -and postimplementation groups were done using the χ ^2^ test. Confidence intervals (CIs) were calculated using the Wilson method. Assuming that errors would be approximately normally distributed, a linear regression model was used to detect linear trends in the prevalences of ceftriaxone and all antibiotic prescriptions over time. Seasonality was adjusted for using harmonic terms with a 1-year period. For the PPS, data were recorded as missing if a patient file was not available at the time the survey was carried out. We assumed that missing records were missing at random and, therefore, were not likely to bias the prevalence estimates. Data were analyzed using the R statistical package, Version 3.6.0 (R Foundation for Statistical Computing, Vienna, Austria).

#### Ethics

The study was approved by the Research Ethics Committees of the University of Malawi, the College of Medicine (P.10/15/1811), and the Liverpool School of Tropical Medicine (15–052RS).

## RESULTS

The records of 203 patients were reviewed in the preimplementation survey (Survey 1). There were 100 records reviewed in the first postimplementation survey (Survey 2) and 200 in the second (Survey 3).

Table 1 shows the characteristics for all included patients. The HIV prevalences were consistent across all 3 surveys (61.2–61.6%), as were recorded ART coverage levels (79.6–81.4%). The median length of a hospital stay and the in-hospital mortality rate did not change pre- and postintervention (Table 1). The rates of confirmed bloodstream infections ranged between 8.4 and 13.1%. The most frequently isolated pathogens were as previously reported from QECH [19], and included *Salmonella* Typhi, *Salmonella* Typhimurium, and *Klebsiella pneumoniae* (Supplementary Results; Table 3).

**Table 1.** Demographic and Outcome Data for Patients Included in the Antibiotic Surveys

	Survey 1	Survey 2	Survey 3
	n = 203	n = 100^a^	n = 200
Age, median (IQR), years	39 (30–53)	33 (25–45)	40 (30–50)
Male, n*/*N (%)	102/203 (50.2)	447/100 (47.0)	103/200 (51.5)
Living with HIV, n*/*N (%)	122/198 (61.6)	49/85(57.6)	118/193(61.1)
	5 unknown	15 unknown	7 unknown
On ART if living with HIV, n*/*N (%)	98/122 (80.3)	39/49 (79.6)	96/118 (81.4)
LOS, median (IQR), days	7 (4–10)	7 (4–9)	7 (5–12)
In-hospital case-fatality, n/N (%)	30/193 (15.5)	17/100 (17.0)	22/172 (12.8)
% (95% CI)	unknown outcome = 10		unknown outcome = 8

Abbreviations: ART, antiretroviral therapy; CI, confidence interval; HIV, human immunodeficiency virus; IQR, interquartile range; LOS, length of stay.

^a^Note that Survey 2 was smaller than Surveys 1 and 3 because of limited personnel available at the time of the survey.

**Figure 1.**
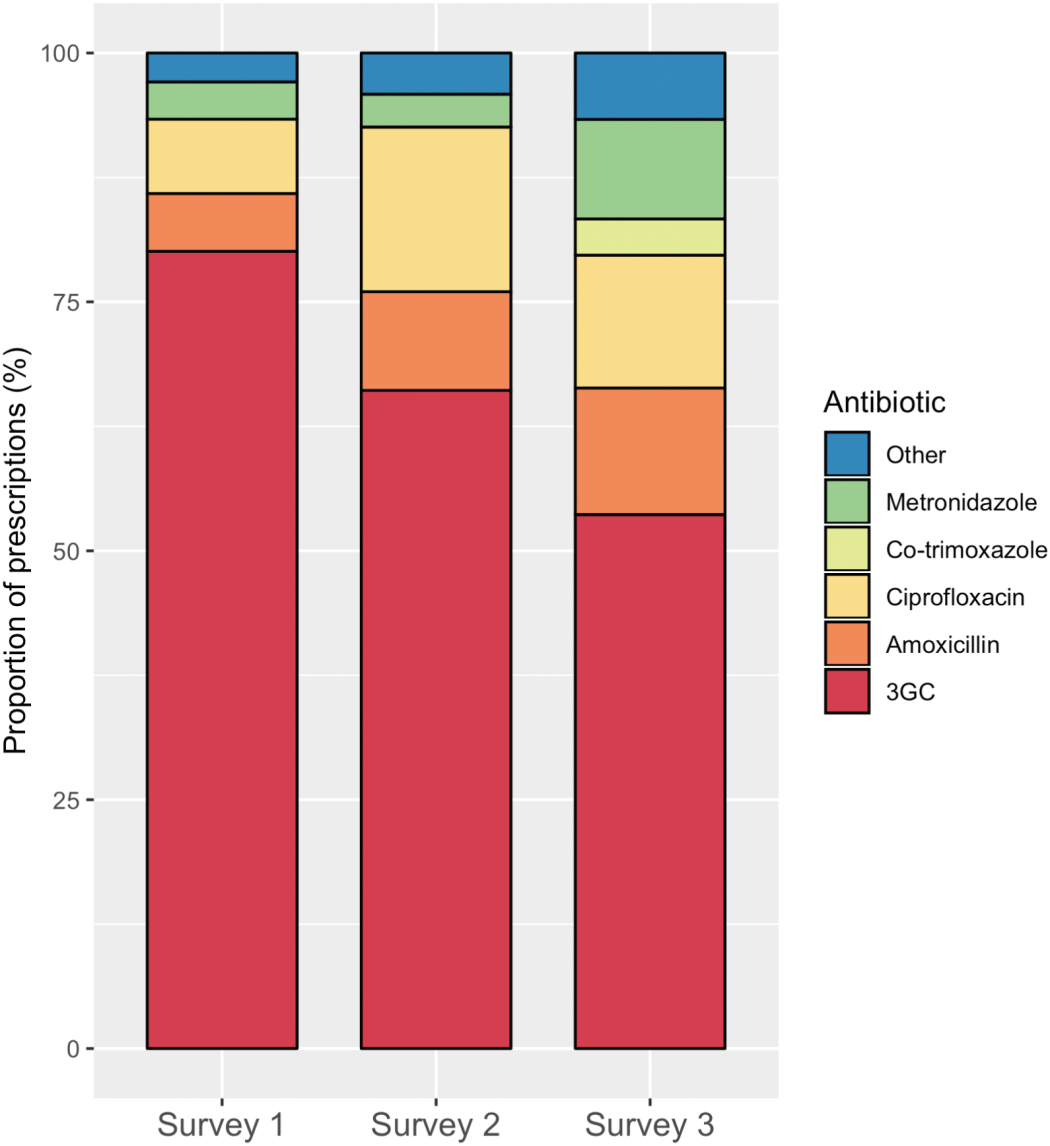
Antibiotic usage pre- and postimplementation, shown as proportions of overall antibiotic prescriptions in the pre- and postimplementation antibiotic surveys. Survey 1 was preimplementation and Surveys 2 and 3 were postimplementation. Abbreviation: 3GC, third-generation cephalosporins.

The clinically suspected focuses of infection, as recorded in the medical notes, were similar across all surveys (Supplementary Results; Figure 1). Respiratory or central nervous system infections were the most common syndromes recorded by admitting teams, whilst nonfocal infections accounted for over 10% of recorded diagnoses. Across the 3 surveys, the proportion of records in which a suspected focus of infection was not recorded prior to antibiotic administration fell from 15.3% (Survey 1) to 6% (Surveys 2 and 3).

**Figure 2.**
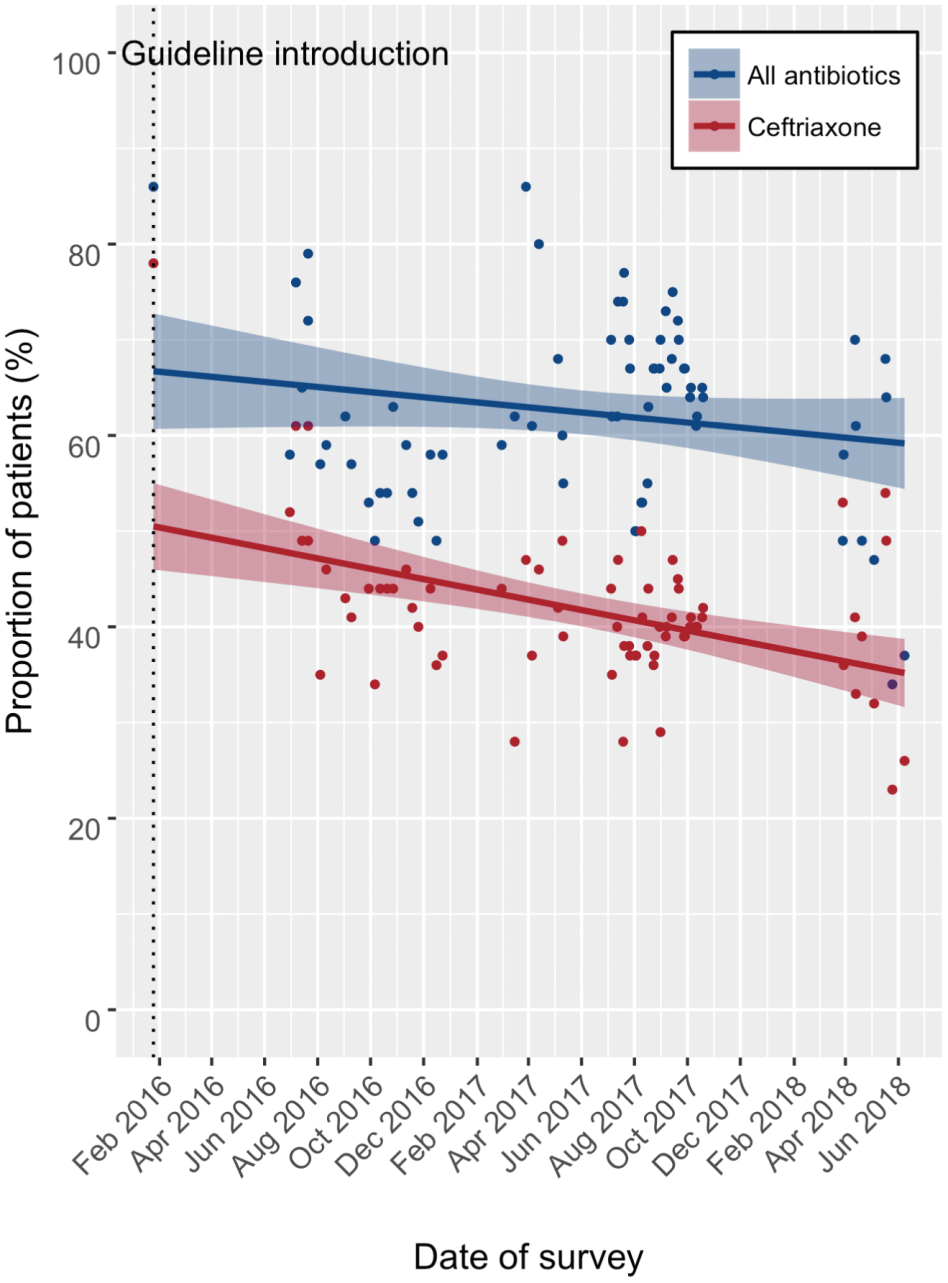
Point prevalence surveys of ceftriaxone and all antibiotics shown pre- and postimplementation. The antibiotic guideline was launched on 30 June 2016 (shown by dotted line) and the study period was from 30 January 2016 to 8 June 2018.

A reduction in 3GC usage was seen after implementation of the stewardship program, as measured by pre- and postimplementation antibiotic surveys (Figure 1; Table 2), antibiotic PPS (Figure 2), and the median duration of antibiotic prescriptions (Figure 1; Table 2).

**Table 2.** Antibiotic Prescriptions on the Medical Wards

	Survey 1			Survey 2			Survey 3		
Antibiotic	**Proportion of Prescriptions, Number** ^**a**^ **%**		**Duration, Days, Median (IQR)**	**Proportion of Prescriptions, Number** ^**a**^ **%**		**Duration, Days, Median (IQR)**	**Proportion of Prescriptions, Number** ^**a**^ **%**		**Duration, Days, Median (IQR)**
3GC^b^	193/241	80.1	5.0 (3.0–8.0)	80/121	66.1	4.0 (2.0–7.0)	177/330	53.6	4.0 (2.0–7.0)
Ciprofloxacin	18/241	7.5	4.0 (3.0–4.0)	20/121	16.5	6.0 (5.0–7.0)	44/330	13.3	3 (1.75–7.25)
Amoxicillin	14/241	5.8	3.0 (2.0–5.0)	12/121	9.9	5.0 (2.5–7.0)	42/330	12.7	3.5 (1.0–6.0)
Metronidazole	9/241	3.7	7.0 (5.0–8.0)	4/121	3.3	3.0 (2.5–3.5)	33/330	10.0	3.0 (2.0–6.0)
Flucloxacillin	3/241	0.9	5.0 (3.0–11.5)	0	0	-	5/330	1.5	6.0 (1.0–15.0)
Erythromycin	2/241	0.8	4.0 (3.0–4.0)	0	0	-	1/330	0.3	1
Benzylpenicillin	1/241	0.4	6.0	1/121	0.8	1	1/330	0.3	12
Co-amoxiclav	1/241	0.4	6.0	1/120	0.8	3	9/330	2.7	2.0 (2.0–3.0)
Doxycycline	0	0	-	2/121	1.7	4.4 (4.25–4.75)	2/330	0.6	5
Gentamicin	0	0	-	1/121	0.8	1	4/330	1.2	1.5 (0–3.5)
Co-trimoxazole	0	0	-	0	0	-	12/330	3.6	4 (2.75–5.25)
All	241	100	4.5 (3.0–7.0)	120	100	5.0 (2.0–7.0)	330	100	4 (2–6)
Total cost^c^	US$1907.04			US$584.78			US$1404.34		
Cost per patient^c^	US$9.39			US$5.85			US$7.02		

Abbreviations: 3CG, third-generation cephalosporin; IQR, interquartile range.

^a^ The denominator is the number of individual antibiotic prescriptions in each survey, not the number of patients. An individual antibiotic prescription was defined as each prescription written on the patient’s chart. If the antibiotic was switched, this was counted as a second prescription.

^b^Cefotaxime was used in place of ceftriaxone during a period of ceftriaxone shortage in 2017.

^c^2017 US Dollars.

The proportion of all antibiotic prescriptions for an intravenous 3GC fell from 193/241 (80.1%) to 177/330 (53.6%; percentage decrease, 26.5%; 95% CI, 18.7–34.1) between the first and last surveys, and the median length of ceftriaxone course was reduced from 5 to 4 days (Table 2). This paralleled an increase in the number of clinician 48-hour reviews of antibiotic prescriptions, as documented in the medical notes. Prior to the stewardship intervention, only 54/241 (22.4%) of prescriptions showed evidence of a 48-hour review, which increased to 242/330 (73.3%) by the final antibiotic survey (percentage increase, 50.9; 95% CI, 43.4–58.4). This and additional key stewardship indicators from the pre- and postimplementation surveys are shown in the Supplementary Results and in Table 4.

The median number of charts included in each PPS was 100 (interquartile range, 94–101). A median of 2 files (interquartile range, 2–4) were recorded as missing in each survey. In the first PPS, 78% of patients on the ward were receiving ceftriaxone and 86% were receiving at least 1 antibiotic of any class. Figure 2 shows the results of 71 PPS carried out over a period of 28 months, with a decline in ceftriaxone prescriptions over the study period (*P* < .01). Although Table 2 shows an increase in ciprofloxacin and metronidazole prescriptions across the 3 prescribing surveys, there was no evidence of a linear trend in the all-antibiotic prevalence over time (*P* = .15; Figure 2). The coefficients of the harmonic terms in the regression model suggested little evidence of a seasonal variation in either ceftriaxone usage (*P* = .19 and *P* = .65 for the sine and cosine terms, respectively) or overall antibiotic usage (*P* = .62 and *P* = .30 for the sine and cosine terms, respectively).

The total costs of antibiotics prescribed to patients in Survey 1, Survey 2, and Survey 3 were US$1907.04, US$584.78, and US$1404.34, respectively. The average costs per patient were US$9.39 (Survey 1), US$5.85 (Survey 2), and US$7.02 (Survey 3; Supplementary Results; Supplementary Table 5). Table 3 shows the mean cost per patient and the total cost estimated from modelling the impact of the stewardship program across the 3 adult medical wards at QECH. The mean cost per patient and the total costs were lower on the TB ward, as many patients are admitted to this ward to receive the initial phase of TB treatment and the costs of anti-TB drugs were not included here. At the patient level, the mean cost across the 3 adult medical wards is projected to fall from US$6.79 per patient to US$5.23 per patient. The total annual cost of providing antibiotics to these 3 wards is projected to fall from US$67 058.14 to US$51 566.60. The total annual cost of delivering the intervention was US$4358.78 (Supplementary Methods).

**Table 3.** Estimated Health Provider Cost of Providing Antibiotics to Medical Inpatients in Queen Elizabeth Central Hospital

	Prestewardship Program	Poststewardship Program
	2017 US$	2017 US$
Mean cost per patient (95% CrI)		
TB ward	5.14 (3.68, 6.59)	4.16 (3.00, 5.31)
Male medical ward	7.31 (6.34, 8.28)	5.90 (5.08, 6.72)
Female medical ward	6.53 (5.54, 7.52)	5.23 (4.45, 6.02)
All medical inpatients	6.79 (6.15, 7.44)	5.23 (4.45, 6.01)
Total cost per annum^a^		
TB ward	4313.74	3491.69
Male medical ward	33 783.43	27 268.02
Female medical ward	25 960.97	20 806.89
All medical inpatients^b^	67 058.14	51 566.60

Abbreviations: CrI, credible interval; TB, tuberculosis.

^a^Based on admissions/year: 840 for TB ward; 4620 for male medical ward; and 3975 for female medical ward.

^b^Total annual cost for all medical inpatients, estimated from summing total annual costs for each ward.

There were 2 rounds of semi-structured interviews conducted: 20 in the pre-implementation period and 21 in the postimplementation period. Due to a high turnover of junior-level doctors on the medical wards, only 1 participant was interviewed both before and after the guideline introduction. The characteristics of interviewees are summarized in the Supplementary Results, Supplementary Table 6. An analysis of the qualitative data identified 6 key themes relating to the stewardship intervention: 3 facilitators (accessibility of information, trust in guideline content, and awareness) and 3 barriers (operational barriers, hierarchical relationships, and rationalized overprescribing). These themes are summarized below and key quotes are presented in Table 4.

**Table 4.** Quotes From Participant Interviews and Observations, Shown By Theme

Theme	Participant	Quote	
1. Accessibility of information	Registrar, postimplementation	1.1	Due to limited knowledge of antibiotics, I would just give a broad-spectrum antibiotic, because to me that makes me well covered. The patient is going to improve … so whatever this patient has he is going to improve. But now at least I am able to sit down and think, what does, what do the guidelines say.
	Registrar, postimplementation	1.2	For UTI, we used to using (*sic*) ciprofloxacin and now they are saying use nitrofurantoin and I keep forgetting the dosing because I’ve never used it my whole life, this is the first time. Yeah, so they do (help).
	Intern, preimplementation	1.3	I remember the time we were switching from cefotaxime to ceftriaxone nobody knew what doses—you may find a meningitis patient given cefotaxime 1g bd, another meningitis patient getting 1g cefotaxime TDS, another bacterial meningitis got 1g od for about 3 days—which is when I saw the patient and changed the dose to the dosage I thought was nice. I feel we sometimes lack the guidance.
	Consultant, preimplementation	1.4	There is so-called “Malawi standard treatment guidelines.” There are some antibiotic guidelines but no proper antibiotic guidelines in Queens that we can use as a facility as such. We do have a medical handbook in our department that we use, and it does help us, it does guide us on what antibiotics we should give, but it’s not very detailed. I feel, it’s not very specific, it’s general, at the end of the day it’s up to the clinician, should I give this antibiotic or not, I feel, because of a lack of a proper guideline, at times patients are started on the strongest antibiotic we have available, that’s ceftriaxone, I find maybe they don’t even meet the criteria to have that antibiotic, but everyone’s on ceftriaxone. I think we don’t have proper guidelines, in short.
	Consultant, postimplementation	1.5	I think the fact that they’ve been made available in electronic forms and also there’s a small booklet which you can carry to the wards. I think that it’s a step in the right direction, I think we should have less excuses for not following the guidelines.
2. Trust in the content	Consultant, postimplementation	2.1	I think we were part of the discussions and we were all consulted and told to make suggestions of the guidelines.
	Consultant, postimplementation	2.2	So I mean essentially the guidelines are based on the data that has been generated over the years in terms of the likely, the commonest organisms that are affecting patients in our setting and …we were all consulted and told to make suggestions.
	Consultant, postimplementation	2.3	They are realistic guidelines … and what they’ve done, is they’ve made sure that most of the drugs that are usually in stock are there, so they are not some fancy drugs that you can hardly find here.
	Registrar, postimplementation	2.4	In terms of coverage, it covers most of the important infections we see in our setting. And in terms of management I think it also gives us alternative in case 1 drug is out of stock there are always alternatives.
3. Awareness and promotion	Consultant, postimplementation	3.1	I think the only notable change that I can comment is on the usage of ceftriaxone, because normally we get a report is it on every Thursday before the ward round on the percentage of ceftriaxone usage in the department, so I think from the figures, from the initial figures and the current figures it seems there has been a significant drop in terms of … usage of ceftriaxone. I think now not many people they are using ceftriaxone so meaning now they are following the guidelines so not giving ceftriaxone to each and every patient.
	Registrar, postimplementation	3.2	Yeah, so, since I think as a department there was quite a lot of awareness and raising awareness that we would have the antibiotic guidelines.
4. Operational barriers	Registrar, postimplementation	4.1	Let’s say there’s amoxicillin, but I’d want to give something slightly more broad spectrum, like augmentin. But there isn’t. And probably the next best thing is ceftriaxone. So sometimes you use an antibiotic which you didn’t necessarily want to use.
	Registrar, preimplementation	4.2	One of them is because the oral drug is out of stock, so the only choice I had was to give a broad spectrum that was IV, but if I had a chance I would have given an oral antibiotic. It has happened so many times, not once.
	Registrar, postimplementation	4.3	For example there should be commitment from management to ensure that even simpler antibiotics should be made readily available because even if broad-spectrum antibiotics only are available and patient has come in with simple, community-acquired pneumonia, people may be tempted to use broad-spectrum antibiotic, because they are only what is available. So, I think there should be a commitment from the management team to ensure that more antibiotics are available.
	Observation, postimplementation	4.4	All the (noninfective) neurological cases viewed this morning had an infective differential. All of these patients had HIV so this is not just speculation, it’s a real risk. Insufficiency of neuroimaging means that the infective cause cannot be ruled out until we get an MRI or the LP or blood culture results get back—all of which will be 5 days.
	Consultant, postimplementation	4.5	When I arrived in 2009, the numbers of patients on our wards was really horrendous … so there will be 1 on the bed, 1 on the floor, all the way into the corridors. So if you had 2 trained nurses per shift it meant that they would be sitting at their desk drawing the antibiotics the whole day. … if they were to do that 4 times a day they did nothing else. So … patients stared getting maybe 1 dose, or 2 doses … but never 4 doses. So we sort of like just slowly drifted towards … once daily antibiotics, ceftriaxone.
5. Hierarchical relationships and prescribing practice	Observation, postimplementation	5.1	There is a palpable power dynamic on this ward round. … Consultant says out loud we should stop ceftriaxone and wrote “CSF normal, stop ceftriaxone.” He did not look at the drug chart and did not cross ceftriaxone off. No-one else on ward round crossed off … and we moved on to the next patient. Certainly, ward round participants don’t seem keen to speak unless directly addressed, maybe they don’t want to cause disruption to the ward round by stopping to cross off antibiotic.
	Medical student, preimplementation	5.2	But as students when you see the files and you see people prescribe amoxicillin, ceftriaxone, you think that’s the way to go because we see people practicing it, but then sometimes when you’re on ward round you see a consultant prescribing an antibiotic which you never know is here.
	Registrar, preimplementation	5.3	I think the interns who come to the department, they see most of the time people are on ceftriaxone, so when they are stuck, they will think that maybe by giving that, they will be off the hook.
6. Rationalized overprescribing	Registrar, preimplementation	6.1	That could be 1 possibility, because if I am certain you can just give an antibiotic which you feel is safe.
	Consultant, postintervention	6.2	And when the patients are perceived to be quite unwell, that’s where the problem of sticking to the guidelines seems to be an issue.
	Consultant, postintervention	6.3	It basically makes people not to think because they have a knee-jerk reaction to everyone who has a fever, to give them ceftriaxone without thinking as to where the focus of infection is, so that you can choose an appropriate antibiotic for the focus of infection. So everyone just gives ceftriaxone as a fall-back position. So it stops people thinking about what is their ideal treatment in this setting.

Abbreviations: bd, twice daily; CSF, cerebrospinal fluid; HIV, human immunodeficiency virus; IV, intravenous; LP, lumbar puncture; MRI, magnetic resonance imaging; od, once daily; tds, three times daily; UTI, urinary tract infection.

### Facilitators

#### Theme 1: Accessibility of Information

The majority of participants in the postimplementation period reported use of the guideline (quotes 1.1–1.2) and the regular consultation of Microguide was observed during ward rounds. This was in marked contrast to the preimplementation interviews, in which participants cited a range of resources used to guide prescribing and difficulty in finding locally relevant information (quotes 1.3–1.4). Junior-level doctors, in particular, perceived that they now had a new resource that would guide them towards narrow-spectrum antibiotics, and valued the ability to access the guideline on their smartphones (quote 1.5).

#### Theme 2: Trust in the Guideline Content

Most participants felt invested in the guideline, noting the department’s involvement in its inception and development (quotes 2.1–2.2). The locally appropriate nature of the guidance was cited as crucial in almost all postimplementation interviews, in particular with regard to antibiotic availability and pathogens (quotes 2.2–2.4).

#### Theme 3: Awareness and Promotion of Stewardship and Antimicrobial Resistance

The process surrounding the introduction of the stewardship intervention was perceived to raise awareness of AMR and of overprescribing within the department. Physicians of all cadres reported thinking more about their antibiotic prescribing practices than before the intervention (quotes 3.1–3.2).

### Barriers

Multiple routes to ceftriaxone overprescribing were identified in the thematic analysis in the pre- and postimplementation phase.

#### Theme 4: Operational Barriers

An overarching theme across interviews and observations was the influence of resources on prescribing practices. Limited access to alternative antibiotics (quotes 4.1–4.3) and comprehensive diagnostics (quote 4.4), as well as an inadequate nursing capacity favoring antibiotics with once-daily dosing regimens (quote 4.5), were frequently cited as reasons for ceftriaxone use and overuse, as perceived by participants.

#### Theme 5. Hierarchical Relationships and Prescribing Practice

Hierarchical relationships between the different cadres of clinical staff shaped prescribing practices. Junior team members invariably took direction from senior colleagues on prescribing, and a rigid hierarchy frequently prohibited junior team members from challenging the prescribing of peers or senior colleagues (quotes 5.1–5.3).

#### Theme 6: Rationalized Overprescribing

Participants frequently described ceftriaxone as the rational choice in the clinical situations they faced, with the risk of undertreating individual patients taking priority over the population-level consequences of antibiotic overuse. Junior doctors considered intravenous antibiotics to be inherently superior to oral, particularly amongst inpatients, where the burdens of HIV and infection are high (quotes 6.1–6.3).

## Discussion

We demonstrate that antimicrobial stewardship is feasible and effective in a low-income country and in a hospital that has no specialist clinical microbiology or ward-level pharmacy service, a limited antibiotic formulary, and no previous ethos of stewardship. A locally appropriate, pragmatic antibiotic guideline using smartphone technology, supported by a simple educational strategy of weekly “reminders,” led to a significant reduction in 3GC usage, an increase in the proportion of 48-hour antibiotic reviews, and a cost savings of over US$15 000 for the medical wards. Critically, the case fatality rates did not change between the pre- and postimplementation surveys, nor on the medical wards in general during the study period.

Unlike in high-income settings, the evidence for successful stewardship in low- and middle-income countries is limited. AMS programs from Cambodia and South Africa have demonstrated substantial impacts. However, whilst resource-constrained, these settings are very different from Malawi, and their stewardship successes relied upon established pharmacy infrastructures [20, 21], specialist microbiology liaisons [22], and a comprehensive formulary [23].

Whilst QECH benefits from sustained access to a quality-assured blood culture service, we highlight multiple operational barriers to implementing stewardship that are distinct to low- and middle-income countries [24–27], but especially a reliance on ceftriaxone due to the clinical context: critically unwell patients, a high burden of infectious disease, and limited diagnostics. Clinical decision-making is not only influenced by guidelines and operational constraints; the social determinants of prescribing must be understood in order to effect a change in practice [28]. Hierarchy and peer pressures shaped antibiotic prescribing decisions in our study, as in other settings [26], but these influences have not previously been explored in Africa, where qualitative studies of stewardship have focused on nursing behaviors [24, 25] and general perceptions of AMR [29]. More in-depth social research should be undertaken to provide a deeper understanding of the mechanisms for changing behavior, if large-scale programs are to be successful.

This study has several limitations. Although the PPS show a significant decline in the prevalence of ceftriaxone usage over the study period, we have limited data from the preimplementation phase. However, previous studies at QECH prior to the intervention showed ceftriaxone prescribing prevalences of 70–84% [19, 30], consistent with our preimplementation findings. Further, we did not engage with an implementation science framework to guide the design of this first-stage qualitative study, which would be a necessary component of a scale-up. We have implemented an AMS program on adult wards, but pediatric and community-level antibiotic usage levels are likely to be major drivers of antibiotic consumption and, thus, AMR [31]. Large-scale stewardship must target these settings next.

We have demonstrated the sustainability of the AMS program over a 28-month period, but measuring its long-term impact, whilst essential, is outside the scope of this study. The potential cost savings achieved by the reduction in antibiotic usage is key to the sustainability of our program, and an antibiotic pharmacist could continue the regular PPS. Involving clinical departments and hospital management in guideline development is fundamental to its sustainability. Our guideline is integrated into departmental teaching and the Microguide application provides a function for real-time feedback from prescribers, allowing regular updates to be incorporated as often as required.

AMS programs in low-income countries are challenged by the dual burden of a high prevalence of severe, often drug-resistant infections alongside insecure, variable access to a range of effective antimicrobials. We demonstrate that stewardship can be adapted to this setting and, by focusing on pragmatic interventions and simple targets, we show the feasibility, acceptability, and cost savings of a stewardship program in Malawi, which was successful in reducing third-generation cephalosporin usage. In doing so, we provide a suitable starting point for expanded, large-scale stewardship interventions in this and other resource-limited settings.

## Supplementary Material

ciaa162_suppl_Supplementary_Figures_1

ciaa162_suppl_Supplementary_Materials

## Notes

***Acknowledgements.*** The authors thank the clinical staff at Queen Elizabeth Central Hospital and the laboratory staff at the Malawi-Liverpool Wellcome Trust Clinical Research Programme.

***Financial support.*** This work was supported by the Malawi-Liverpool Wellcome Trust Major Overseas Programme Core Grant (grant number 206545/Z/17/Z). R. L. is supported by a Wellcome Trust Clinical PhD Fellowship (University of Liverpool block award grant number 203919/Z/16/Z).

***Potential conflicts of interest*.** D. G. L. received grants from the Wellcome Trust, during the conduct of the study. All other authors report no potential conflicts. All authors have submitted the ICMJE Form for Disclosure of Potential Conflicts of Interest. Conflicts that the editors consider relevant to the content of the manuscript have been disclosed.
