# Supplementary figures and images for: Sustained Reduction in Third-generation Cephalosporin Usage in Adult Inpatients Following Introduction of an Antimicrobial Stewardship Program in a Large, Urban Hospital in Malawi
Source: Clin Infect Dis. 2020 Feb 15;71(9):e478–86. doi: 10.1093/cid/ciaa162 (PMC7713689; doi:10.1093/cid/ciaa162)

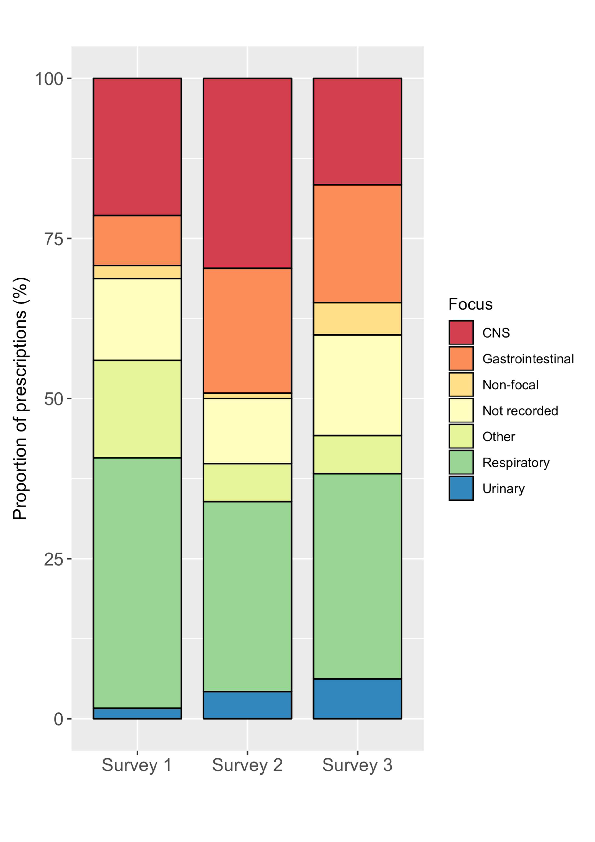

Supplement: ciaa162_suppl_Supplementary_Figures_1 [file ciaa162_suppl_supplementary_figures_1.png]
