# Supplementary material for: Sustained Reduction in Third-generation Cephalosporin Usage in Adult Inpatients Following Introduction of an Antimicrobial Stewardship Program in a Large, Urban Hospital in Malawi
Source: Clin Infect Dis. 2020 Feb 15;71(9):e478–86. doi: 10.1093/cid/ciaa162 (PMC7713689; doi:10.1093/cid/ciaa162)
Supplement: ciaa162_suppl_Supplementary_Materials [file ciaa162_suppl_supplementary_materials.docx]

**Supplementary Materials**

**1. Supplementary Methods**

The study was developed by RL, NAF, JM and MN and the protocol approved by the COMREC and the LSTM Research Ethics Committees. The guideline was developed by a team of senior clinicians, pharmacists and nurses at Queen Elizabeth Central Hospital (QECH) led by RL and subsequently approved by the Department of Medicine and the Department of Emergency Medicine at QECH. The AMS team was then led on a day to day basis by RL.

**Setting**

Malawi is classified as low-income by the World Bank, ranking 149^th^ out of 205 economies (2018 GDP of USD 7.065 Billion) [1]. Malawi’s healthcare spending as a share of GDP is low, averaging around 3% since 2012 [2]. QECH provides free healthcare to Blantyre and the surrounding districts, plus tertiary care to Malawi’s Southern region. It has 1300 beds, frequently operating above capacity. Adult patients attending QECH must be referred from a primary health centre, or from another hospital if tertiary care is being sought. Patients are triaged 24-hours a day in the Adult Emergency and Trauma Centre (AETC) and either discharged or referred to the appropriate speciality team (medicine, surgery or obstetrics and gynaecology). The Department of Medicine runs two single-sex medical wards, each with an official capacity of about 60 beds, but these wards are frequently oversubscribed with patients nursed on floor mattresses between bed spaces and in corridors.

**Antibiotic prescribing surveys**

Inpatient records were reviewed to collect data on key stewardship quality indicators, which aim to improve antibiotic prescribing in inpatient settings. Guidelines on appropriate stewardship indicators for Malawi are not available, so we adapted standardised indicators from international guidelines [3, 4]. Following discussion with QECH physicians and hospital pharmacists, we selected quality indicators that would be relevant to local practice. These are defined in Supplementary Table 1, and results from the surveys are shown in Supplementary Results, Table 3. These targets were chosen for a number of reasons. Firstly, it was critical that our intervention did no harm through restricting antibiotics at the front door in a context where severe bacterial infection is common, point of care diagnostics are lacking and triage is variably implemented, therefore maintaining appropriate access to potentially life-saving medicine was key. Further, the lack of 48-hour review was identified as a major issue in the first antibiotic prescribing survey. Lastly, the study team identified these as simple measurable targets well-suited to the Malawi setting.

Several indicators were not included because they cannot be applied to the QECH setting. For example, adjustment of antibiotic dosing regimens to renal function and therapeutic drug monitoring are evidence-based quality indicators used in some settings [3], but these blood tests are not routinely available in Malawi.

Patients for the antibiotic surveys were recruited retrospectively. The files of patients who had been discharged or died were reviewed and the patient included if they had received at least one antibiotic for more than 24-hours. The patient records and prescribing charts were used to complete standardised data capture tools which recorded demographic and clinical information, as well as details of each antibiotic prescription. Information on each antibiotic prescription was recorded and the resulting denominator was the number of individual antibiotic prescriptions in each survey. The data capture tools used are attached as the final pages to these Supplementary Materials.

| Supplementary Table 1. Definitions of Stewardship Quality Indicators | |
| --- | --- |
| **Stewardship indicator** | **Definition** |
| 48-hour antibiotic review | Evidence that a prescription had been reviewed by a clinician within 48-hours of its start-date. Evidence of review was defined as a comment in the medical notes on whether to continue, stop or change the antibiotic, or as evidence of a stop or change on the prescription chart. |
| More than 7-days of antibiotic treatment | Prolonged duration of treatment (a prescription continued for 8-14 days). |
| More than 14-days of antibiotic treatment | Prolonged duration of treatment (a prescription continued beyond 14 days). |
| More than three antibiotics concurrently | Overprescribing and concurrent use of three or more antibiotics in the same patient on the same day for at least 48-hours. |
| Redundant cover | Concurrent administration of two or more antibiotics with duplicate spectra of activity, in the same patient, on the same day for at least 48-hours. |

The same data capture tools were used across all three surveys and staff were trained in the use of these tools. Data were entered in accordance with Good Clinical Practice (GCP) guidelines in which the study team were trained. Data were captured anonymously, using structured TeleForm paper forms (OpenText, Waterloo, Canada). Forms were checked for completion and scanned by the study PI (RL) on a weekly basis. Variable values are automatically extracted by the TeleForm system and data queries generated for missing or invalid values were resolved by manually reconciling with the forms, discussion with the data collecting team and reverting to the patient records where necessary.

**Antibiotic guideline**

The guideline development team was led by RL, the study PI who is a specialist in infectious diseases and who worked as a consultant in the Department of Medicine at QECH. The team included a consultant in clinical microbiology, consultants from the Department of Medicine and the Department of Emergency Medicine, and nursing and pharmacy representatives. Three guideline development meetings were held, each lasting an afternoon. At the first guideline development meeting, the results of the first prescribing survey were presented by RL. The consortium decided based on these results, that the importance of a 48-hour review should be a clear message in the guideline. Subsequent meetings were used to draft and review guideline content. As there was no existing departmental guideline to adapt, the contents of each section were decided in these meetings, by a consensus discussion between the clinicians, microbiologists and pharmacists present. If a national guideline existed, for example, for treatment of meningitis or pneumonia, it was reviewed and adapted to the new guideline, taking into account local availability of antibiotics and knowledge of bacterial susceptibility patterns. The guideline was then submitted to both clinical departments (Medicine & Emergency Medicine) for further comment prior to its adoption.

The introductory chapter of the guideline included a section on the importance of 48-hour antibiotic review and each prescribing section stressed the importance of prescription duration as well as review and step-down of antibiotics.

Once the guideline was complete, the results of the first prescribing survey and information about how to access Microguide were presented to the Departments of Medicine and Emergency Medicine during several routine departmental clinical and educational activities, including daily morning meetings and intern and medical student teaching sessions. Senior clinicians encouraged prescribers to refer to the guideline on ward rounds and checked that members of their team were using it.

**Point prevalence surveys (PPS)**

PPS were carried out by two trained study field-workers. On the medical wards, patient records are kept on the end of each bed, so each bed was approached systematically and every available prescription chart reviewed. If a file was not available after searching on the ward, it was recorded as missing. Data were collected on paper forms. For each file, the field worker recorded if the patient was currently on at least one antibiotic (Yes/No) and if the patient was currently on ceftriaxone (Yes/No). At the end of the survey, the field worker recorded the total number of inpatients admitted to the ward at the time of the survey, including those who were not physically present and those whose files were missing. Each survey took approximately 2 hours to complete. The results of the survey were presented verbally, by the study PI at the next departmental morning meeting, prior to a ward round and always in the same week of the survey. The department were informed what proportion of patients were on ceftriaxone and what proportion were on at least one antibiotic and at the same time, reminded to review their prescriptions.

**Qualitative methods**

A qualitative methodology was selected because it provided participants with an opportunity to reflect on and articulate their experiences in their own words [5]. Specific methods were semi-structured interviews and direct observation. The observations were led by AW, and involved spending time over a 6-week period on ward rounds, and during morning meetings and hand-over. These methods allowed for the exploration of deductive themes which were seen in the secondary data, but also in the inductive construction of new themes which emerge as important to the lived-experience of the participants.

Interviews were conducted by a team of three clinicians (PB, AW, SH) and one field-worker. None of the team who conducted the interviews we involved in the implementation phase. Each team member received training in qualitative research methods and the team were supported by experienced social scientists (EM, RT). A discussion guide was used to guide the interviews and was updated iteratively to include new themes and concepts as they were identified. For the semi-structured interviews, participants were purposively sampled based on their role as prescribers on the hospital wards. Junior and senior members of medical department were included in the sample to ensure a range of perspectives. The data collected in the interviews was also triangulated through the observations that took place following the implementation of the intervention. Interviews were audio-recorded, with written informed consent of the participants, and conducted in English if carried out by doctors and in Chichewa if carried out by field-workers. The observations centred on the adult medical wards at QECH in July 2018 (AW). AW attended ward rounds, clinical hand-over meetings and sent time observing day-to-day clinical practice on the wards. The central focus was on prescribing practices of clinicians and how this practice was enacted day-to-day on the wards. The qualitative analysis drew on a thematic content analysis approach, with a coding scheme developed based on participants responses and the fieldnotes [5]. The analysis was carried out through an iterative process with preliminary analysis informing the next round.

**Cost analysis**

We estimated the annual economic cost of implementing the Antibiotic Stewardship Intervention from the healthcare provider perspective. We excluded all research related costs. The intervention consisted of: (1) weekly point prevalence surveys across the three wards; (2) feedback of findings to the clinicians at their weekly team meeting; (3) a smartphone application (Microguide, <http://www.microguide.eu/>); and (4) distribution of pocket-sized booklets and posters in key prescribing areas. We estimated the annual costs for these components of the intervention in 2017 US Dollars (Supplementary Table 2).

In our study, a field worker undertook the point prevalence surveys and feedback findings to the clinicians. The field worker took approximately 2 hours to conduct the survey for each ward and a further 1 hour to collate the data. They took approximately 10 minutes of the weekly clinical team meeting to feedback the findings. Under real-world implementation we assumed this task would be undertaken by pharmacist technician. We took a conservative view and assumed the pharmacist would be employed full-time on the service. We included the salary costs of all the clinicians attending the weekly teams meetings but excluded any overhead costs of holding the meeting. The licence for the MicroGuide application lasts three years and their costs were annualized using a 3% discount rate.

**Supplementary Table 2: Annual Economic Cost of the Stewardship intervention**

| **Cost category** | **2017 US Dollars** |
| --- | --- |
| Pharmacist | 3404.45 |
| Clinician team meetings | 177.95 |
| MicroGuide application | 571.70 |
| Booklets and posters | 204.68 |
| **Total** | **4,358.78** |

We modelled the impact of the stewardship intervention on the mean costs per participant and the total annual cost of providing antibiotics across the three wards. We used antibiotic consumption data from a cohort of medical inpatients admitted to these wards in 2014 [6]. In this previous study we had collected individual-level data on the ward admitted to, the types of antibiotics given, as well as the route of administration, dosage and number of doses given from admission to discharge in 2014. We used this cohort to estimate the direct health provider cost per participant for the pre-implementation phase. A scenario where no antibiotic stewardship intervention was being delivered.

We used the percentage change in prescriptions of the different antibiotics observed from the pre-implementation prescribing survey to the post-implementation survey to model the likely antibiotic usage and therefore the cost per participant for the post-implementation phase. A scenario where antibiotic stewardship intervention had been implemented.

We used the annual number admissions to each of these three wards and the mean cost per participant to estimate the total cost per annum incurred from providing antibiotics in each of the three wards. We used the international market price of drugs [7] and converted into 2017 US Dollars using World Bank conversion factors [8].

**2. Supplementary Results**

Supplementary Table 3. Blood culture data for patients included in the antibiotic surveys

|  | Survey 1 | Survey 2 | Survey 3 |
| --- | --- | --- | --- |
| Blood culture taken  n/N (%) | 88/203(43.3) | 38/100 (38) | 107/200 (53.5) |
| Blood culture confirmed BSI  n/N (%) | 10/88 (11.4) | 5/38 (13.1) | 9/107 (8.4) |
| Organism isolated n/N  *Salmonella* Typhimurium  *Salmonella* Typhi  *Klebsiella pneumoniae*  *Escherichia coli*  *Streptococcus pneumoniae*  Staphylococcus aureus  *Cryptococcus neoformans* | 4/10  3/10  2/10  1/10  1/10  0  0 | 0  1/5  0  0  2/5  1/5  1/5 | 3/9  1/9  2/9  1/9  0  0  2/9 |

**Abbreviation:** BSI, bloodstream infection

| Supplementary Table 4. Stewardship indicators from the antibiotic surveys | | | |
| --- | --- | --- | --- |
|  | Survey 1 | Survey 2 | Survey 3 |
| **Stewardship indicator** | n/N (%) | n/N (%) | n/N (%) |
| 48-hour antibiotic review^a^ | 54/241 (22.4) | 58/121 (47.9) | 242/330 (73.3) |
| More than 7-days of antibiotic treatment^a^ | 58/241 (24.0) | 21/121 (17.4) | 62/330 (18.7) |
| More than 14-days of antibiotic treatment^a^ | 14/241 (5.8) | 0/121 | 13/330 (3.9) |
| More than three antibiotics concurrently | 0/203 | 0/100 | 0/200 |
| Redundant cover^b^ | 10/203 (4.9) | 2/100 (2.0) | 18/200 (9.0) |

^a^ Denominator is total number of antibiotic prescriptions in the survey

^b^ Denominator is number of patients in the survey

| Supplementary Table 5: Antibiotic consumption and cost | | | | | | |
| --- | --- | --- | --- | --- | --- | --- |
|  | **Survey 1 (n=203)** | | **Survey 2 (n=100)** | | **Survey 3 (n=200)** | |
| Antibiotic | **Total doses** | **Quantity (mg)** | **Total doses** | **Quantity (mg)** | **Total doses** | **Quantity (mg)** |
| 3GC | 1934 | 3,582,042 | 543 | 1,059,000 | 1625 | 2,124,010 |
| Ciprofloxacin | 162 | 8600 | 175 | 102750 | 451 | 531,105 |
| Amoxicillin | 211 | 108500 | 131 | 119500 | 272 | 377000 |
| Metronidazole | 183 | 86100 | 28 | 11200 | 274 | 361675 |
| Flucloxacillin | 96 | 48000 | 0 | 0 | 86 | 79600 |
| Erythromycin | 52 | 26000 | 0 | 0 | 4 | 361675 |
| Benzylpenicillin | 28 | 33600 | 1 | 1200 | 37 | 44400 |
| Co-amoxiclav | 21 | 13125 | 9 | 5625 | 58 | 86500 |
| Doxycycline | 0 | 0 | 17 | 1700 | 27 | 30,000 |
| Gentamicin | 0 | 0 | 1 | 2400 | 18 | 17750 |
| Co-trimoxazole | 0 | 0 | 0 | 0 | 100 | 144540 |
| Average cost of antibiotics given per participant  (2017 US$) | **US$9.39** | | **US$5.85** | | **US$7.02** | |

| Supplementary Table 6. Characteristics of interview participants | | |
| --- | --- | --- |
| Characteristic | Interview participants, n (%)  Pre-implementation | Interview participants, n (%)  Post-implementation |
| Grade of participant  Consultant  Registrar  Medical officer  Intern  Medical student  Clinical officer | 4  2  5  2  3  4 | 7  9  0  2  2  1 |
| Sex  Male  Female  Not recorded | 11  9  0 | 10  5  6 |
| Years worked or studied in medical department  <1  1-4  5-10  >=10 | 6  9  3  2 | 6  9  3  3 |


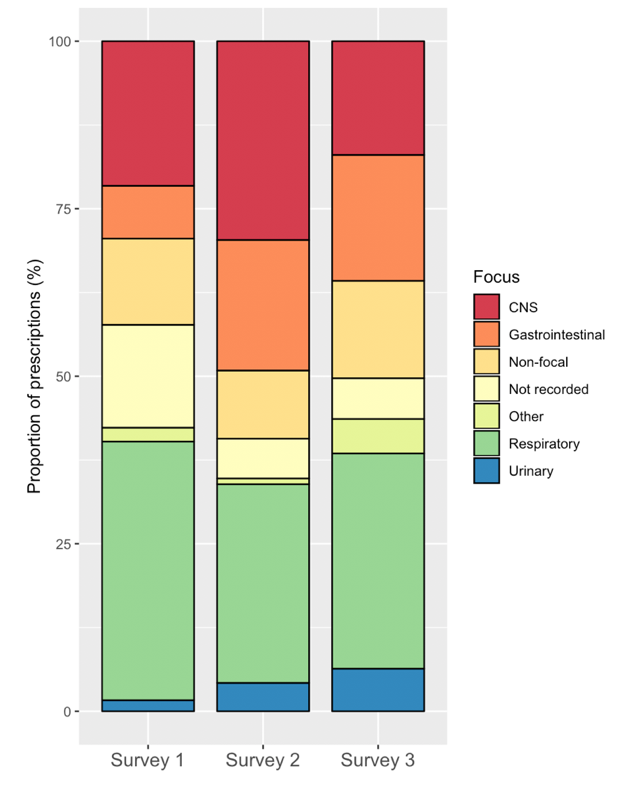


**Supplementary Figure 1.** Clinically suspected focus of infection prompting each antibiotic prescription. CNS=central nervous system

**3. Data Capture Tools**

**References**

1 World Bank. <https://datahelpdesk.worldbank.org/knowledgebase/articles/906519-world-bank-country-and-lending-groups>

2 UNICEF. 2018/19 Health Budget Brief. Towards Universal Health Coverage (UHC): Acheiving SDG 3 in Malawi. 2018. Available at <https://www.unicef.org/esa/sites/unicef.org.esa/files/2019-04/UNICEF-Malawi-2018-Health-Budget-Brief.pdf>. Accessed Jan 4th 2020.

3 Schuts EC, Hulscher M, Mouton JW, et al. Current evidence on hospital antimicrobial stewardship objectives: a systematic review and meta-analysis. Lancet Infect Dis **2016**; 16(7): 847-56.

4 Antimicrobial Stewardship Toolkit for Secondary Care: Start Smart – then Focus Available at: <https://www.gov.uk/government/publications/antimicrobial-stewardship-start-smart-then-focus>.

5 Green J, Thorogood N. Qualitative methods for health research. Sage; 2009.

6 Maheswaran H, Petrou S, MacPherson P, et al. Cost and quality of life analysis of HIV self-testing and facility-based HIV testing and counselling in Blantyre, Malawi. BMC Med **2016**; 14: 34.

7 Ahoyo AT, Baba-Moussa L, Anago AE, et al. [Incidence of infections dues to Escherichia coli strains producing extended spectrum betalactamase, in the Zou/Collines Hospital Centre (CHDZ/C) in Benin]. Med Mal Infect **2007**; 37(11): 746-52.

8 Frank T, Gautier V, Talarmin A, Bercion R, Arlet G. Characterization of sulphonamide resistance genes and class 1 integron gene cassettes in Enterobacteriaceae, Central African Republic (CAR). J Antimicrob Chemother **2007**; 59(4): 742-5.
